# Supplementary material for: Comprehensive In Silico Characterization and Expression Pro-Filing of DA1/DAR Family Genes in Brassica rapa
Source: Genes (Basel). 2022 Sep 2;13(9):1577. doi: 10.3390/genes13091577 (PMC9498896; doi:10.3390/genes13091577)
Supplement: Supplementary file 1 [file genes-13-01577-s001.zip › Figure S1.pdf]

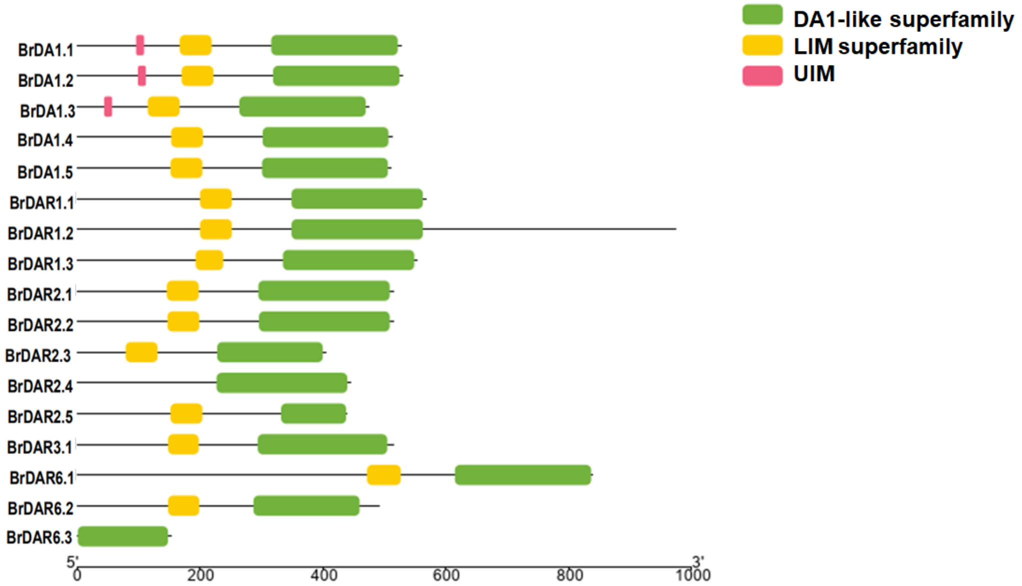

Figure S1. Protein structure analysis to highlight the presence of DA1-like domain in all family members of BrDA1&DARs.
